# Gestational Weight Gain by Maternal Pre-pregnancy BMI and Childhood Problem Behaviours in School-Age Years: A Pooled Analysis of Two European Birth Cohorts

**DOI:** 10.1007/s10995-020-02962-y

**Published:** 2020-06-17

**Authors:** Elena C. Tore, Evangelia E. Antoniou, Renate H. M. de Groot, Marij Gielen, Roger W. L. Godschalk, Theano Roumeliotaki, Luc Smits, Taunton R. Southwood, Marc E. A. Spaanderman, Nikos Stratakis, Marina Vafeiadi, Vaia L. Chatzi, Maurice P. Zeegers

**Affiliations:** 1 Department of Complex Genetics, Care and Public Health Research Institute, Maastricht University, 6200 MD Maastricht, The Netherlands; 2 Institute of Applied Health Research, College of Medical and Dental Sciences, University of Birmingham, Birmingham, B15 2TT UK; 3 Welten Institute, Research Centre for Learning, Teaching, and Technology, Open University of the Netherlands, 6419 AT Heerlen, The Netherlands; 4 Department of Complex Genetics, School for Nutrition and Translational Research in Metabolism, Maastricht University, 6200 MD Maastricht, The Netherlands; 5 Department of Pharmacology and Toxicology, School for Nutrition and Translational Research in Metabolism, Maastricht University, 6200 MD Maastricht, The Netherlands; 6 Department of Social Medicine, Faculty of Medicine, University of Crete, 71003 Heraklion, Greece; 7 Department of Epidemiology, Care and Public Health Research Institute, Maastricht University, 6200 MD Maastricht, The Netherlands; 8 Institute of Child Health, University of Birmingham, Birmingham, B15 2TT UK; 9 Department of Obstetrics and Gynaecology, School for Oncology and Developmental Biology, Maastricht University, 6200 MD Maastricht, The Netherlands; 10 Keck School of Medicine, University of Southern California, Los Angeles, CA 90032 USA

**Keywords:** Gestational weight gain, Pre-pregnancy BMI, Internalizing, Externalizing, Problem behaviours

## Abstract

**Objectives:**

Maternal pre-pregnancy weight is known to affect foetal development. However, it has not yet been clarified if gestational weight gain is associated with childhood behavioural development.

**Methods:**

We performed a pooled analysis of two prospective birth cohorts to investigate the association between gestational weight gain and childhood problem behaviours, and the effect modification of maternal pre-pregnancy BMI. In total, 378 mother–child pairs from the Maastricht Essential Fatty Acids Birth cohort (MEFAB) and 414 pairs from the Rhea Mother–Child cohort were followed up from early pregnancy to 6–7 years post-partum. At follow up, parents assessed their children’s behaviour, measured as total problems, internalizing and externalizing behaviours, with the Child Behaviour Checklist. We computed cohort- and subject-specific gestational weight gain trajectories using mixed-effect linear regression models. Fractional polynomial regressions, stratified by maternal pre-pregnancy BMI status, were then used to examine the association between gestational weight gain and childhood problem behaviours.

**Results:**

In the pre-pregnancy overweight/obese group, greater gestational weight gain was associated with higher problem behaviours. On average, children of women with overweight/obesity who gained 0.5 kg/week scored 25 points higher (on a 0–100 scale) in total problems and internalizing behaviours, and about 18 points higher in externalizing behaviours than children whose mothers gained 0.2 kg/week. Inconsistent results were found in the pre-pregnancy normal weight group.

**Conclusions for Practice:**

Excessive gestational weight gain in women with pre-pregnancy overweight/obesity might increase problem behaviours in school-age children. Particular attention should be granted to avoid excessive weight gain in women with a pre-pregnancy overweight or obesity.

**Electronic supplementary material:**

The online version of this article (10.1007/s10995-020-02962-y) contains supplementary material, which is available to authorized users.

## Significance

*What is already known on this subject?*: Strong evidence suggests that maternal weight before pregnancy may be associated with foetal development. However, little is known about the consequences of inadequate gestational weight gain; recent studies suggest that there might be an association with offspring’s neurocognitive and behavioural development.

*What does this study add?*: Children born to women with pre-pregnancy overweight/obesity who gained 0.5 kg/week during pregnancy showed higher scores in all three assessed behaviour scales, compared to children whose mothers gained 0.2 kg/week. Conversely, no clear association appeared in children born to women with a pre-pregnancy weight in the normal range.

## Introduction

Excessive gestational weight gain (GWG) is a global burden that may have serious consequences on children’s physical and psychological development, especially when combined with a pre-existing overweight status (Institute of Medicine and National Research Council, 2009; Van Lieshout, 2013; World Health Organization, 2016).

An important aspect of childhood psychological development concerns problem behaviours, a group of psychopathological disorders that affects stress reactivity. Internalizing behaviours are typified by anxious and depressive traits, whereas aggressiveness and hyperactivity characterise externalizing behaviours (Achenbach & Resorta, 2001). Early-onset problem behaviours are deemed precursors of several adverse outcomes in later life, including psychiatric disorders and delinquency (e.g., Ferdinand et al., 2004; Reef, van Meurs, Verhulst, & van der Ende, 2010).

To date, only one study has investigated the influence of GWG on childhood problem behaviours, reporting no association (Pugh et al., 2016). Conversely, other studies found poor childhood behavioural outcomes were associated with excessive GWG when combined with pre-pregnancy overweight/obesity (Aubuchon-Endsley et al., 2017; Rodriguez et al., 2008). This study, therefore, aimed to examine the association between GWG and problem behaviours in school-age children. The possible effect modification of maternal pre-pregnancy body mass index (BMI) status was assessed, based on previous evidence of the correlation between GWG and pre-pregnancy weight (Institute of Medicine and National Research Council, 2009).

## Methods

### Study Participants

The Maastricht Essential Fatty Acid Birth (MEFAB) cohort is a prospective birth cohort established in the South of Netherlands (van der Wurff et al., 2015). Between 1989 and 1995, 1334 pregnant women were recruited during their first antenatal visit. After excluding all women with cardiovascular, neurological, renal or metabolic conditions and those who did not provide blood samples for fatty acid assessment, 1203 women were included in the cohort. Of these, 750 were eligible for the 7-year follow-up evaluation.

The Rhea Mother-Child Cohort recruited pregnant women during their first-trimester ultrasound examinations in Crete, Greece, between 2007 and 2008. Eligible women were resident of the Heraklion region and did not present any communication disorder. A total of 1363 singleton pregnancies were followed-up until delivery, as previously described (Chatzi et al., 2017).

To meet the inclusion criteria for this study, participants had to attend a minimum of two prenatal visits during which body weight was measured at least once and provide complete information on child behaviour. Consequently, this study included a total of 378 mother–child pairs from MEFAB (50.4% of participants eligible for the follow up) and 414 from Rhea (30.4% of participants followed-up until delivery).

In accordance with the Declaration of Helsinki, the MEFAB study was approved by the Medical Ethics Committee, University Hospital, Maastricht/ Maastricht University, while the Rhea study was approved by the Ethics Committee of the University Hospital in Heraklion. Written informed consent was obtained from all participants included in the study.

Results of the non-response analyses are presented in the Online Resource 1. The mothers of children with follow-up data were less likely to have overweight/obesity in MEFAB (27.25% vs. 34.20%), although only a small difference was observed in the median early pregnancy BMI (22.90 (21.49, 25.23) vs. 23.46 (21.22, 26.30)). Children included in the Rhea study were more likely to have highly educated parents (40.58% vs. 30.78%) and mothers who were less likely to smoke (17.52% vs. 22.78%). In both cohorts, children with follow-up data weighted about 100 g more at birth than children without follow-up data (MEFAB: 3304.09 g (520.98) vs. 3205.14 g (583.27); Rhea: 3213.18 g (453.36) vs. 3121.06 g (488.82)). Other differences were of minor entity and not expected to affect participation rate.

### Weight in Pregnancy

In MEFAB, hospital staff measured women’s weight in four occasions during pregnancy: at study entry (median; interquartile range (IQR): week 10.14; 8.29, 12.29), during the second (week 21.86; 21.00, 22.86) and third study visits (week 32; 31.43, 32.57), and at delivery (39.43 weeks; 38.29, 40.43). In Rhea, trained midwives measured women’s weight during clinical visits in the first (week 12; 11, 13) and third (week 32; 30, 35) trimesters, while data on women’s weight at delivery was self-reported and collected during telephone interviews conducted 8–10 weeks after giving birth (final gestational age: week 38; 38, 39).

### Pre-pregnancy BMI

In MEFAB, pre-pregnancy BMI (kg/m^2^) was calculated using the measured first trimester weight as a proxy of weight before conception, since no information was recorded regarding pre-pregnancy weight. In Rhea, given the relatively late recruitment (median: week 12), information on self-reported pre-pregnancy weight, collected at study entry, was used to compute pre-pregnancy BMI.

Due to a limited number of women falling into the underweight (BMI < 18.5; n = 25, 3.05%) and obese (BMI ≥ 30; n = 81, 9.88%) pre-pregnancy BMI categories, pre-pregnancy BMI status was computed as normal (BMI < 25 kg/m^2^) vs. overweight/obese (BMI ≥ 25 kg/m^2^).

### Child Problem Behaviours

The Child Behaviour Checklist (CBCL) 4/18 and its revised version, the CBCL 6/18, were used in MEFAB and Rhea, respectively, to assess children’s problem behaviours as perceived by their parents (Achenbach & Edelbrock, 1983; Achenbach & Resorta, 2001). The CBCL has demonstrated good psychometric properties and reliability (Achenbach & Resorta, 2001). Both versions of the CBCL forms have been validated for use in both Dutch and Greek populations (De Groot, Koot, & Verhulst, 1994; Roussos et al., 1999). This study assessed the three CBCL broadband scales: total problems, internalizing and externalizing behaviours. To allow comparability between studies, age-standardized T-scores (with a mean of 50 and a standard deviation of 10) were used. T-scores can range from 0 to 100; high values (i.e., above 63) indicate clinical levels of symptomatology.

### Statistical Analysis

#### Computation of GWG Trajectories (Analyses Stratified by Cohort)

To increase modelling precision, this step of the analysis included all women with available information on at least one measure of gestational weight and at least two measures of gestational age at which weight (or other data) was collected, independently of the availability of follow-up data (n = 1227 in MEFAB and n = 1353 in Rhea; median (IQR) number of measurements per woman: 4 (4, 4) in MEFAB, 3 (2, 3) in Rhea; percentage of women with one weight measurement: 0.24% in MEFAB, 6.95% in Rhea. Additional information is provided in the Online Resource 2). The linearity of the association was explored in each cohort separately; no evidence of deviation from linearity was found. Mixed-effect linear regression models with two levels (i.e., random intercepts for participants and random slopes for measurement occasion) were, then, used to model maternal weight during pregnancy against gestational age. The best linear unbiased predicted slope of gestational weight was obtained for each woman and used as the exposure in the subsequent step of the analyses (Chen et al., 2015). In this study, we refer to the predicted slope of gestational weight as *weekly gestational weight gain* (*wGWG*), as it represents the average weekly increment in weight during pregnancy.

#### Multivariable Regressions (Pooled Analyses)

The associations between wGWG and childhood problem behaviours were assessed with multivariable regression analyses with the best-fitting fractional polynomials of wGWG, since these associations did not follow a linear pattern. Interaction between maternal pre-pregnancy BMI status and wGWG were tested for all outcomes. Furthermore, given the role of sex on prenatal brain development (e.g., Reinius & Jazin, 2009)), the interaction between wGWG and children’s sex was evaluated. Subsequent analyses were stratified based on the effect modifier’s categories in case of statistically significant interactions.

We used a Directed Acyclic Graph (Online Resource 3; (Textor, van der Zander, Gilthorpe, Liśkiewicz, & Ellison, 2016) to identify the covariates to control for. The final covariate set included: maternal age, smoking and alcohol consumption, parental education and parity. In addition, maternal first trimester (MEFAB) or pre-pregnancy (Rhea) weight, children’s age at assessment and a cohort indicator variable were adjusted for. The children’s sex was controlled for in the non-stratified analyses (i.e., those for which a significant interaction with children’s sex was not found).

For ease of interpretation, we used the MIMRGNS command (Klein, 2014) in Stata to predict problem behaviour scores at the 5th, 25th, 50th, 75th and 95th percentiles of wGWG, while keeping constant all other variables included in the model.

To increase the sample size and reduce the bias due to missing values, multiple imputation of missing covariate data was performed using chained equations where 50 completed datasets were generated, separately for the two cohorts (White, Royston, & Wood, 2011). An imputation model including all exposures, outcomes, covariates and additional auxiliary variables was constructed. Auxiliary variables comprised maternal height, subject-specific mean weight in pregnancy, birth weight, gestational age, pregnancy outcomes, children’s BMI at follow-up, breastfeeding status and day-care attendance.

Several sensitivity analyses were performed to assess the robustness of our results. First, we excluded women who gave birth before week 37 of pregnancy, since a preterm birth might influence both wGWG and child development. Second, we included only women with complete information on weight during pregnancy, to rule out the possibility that the group of women with fewer weight measurements differed from the group with complete data. Third, we repeated the analyses in each cohort separately to evaluate potential heterogeneity. Fourth, we additionally controlled for breastfeeding and day-care attendance, since these might independently influence the outcomes. Fifth, we repeated the analyses excluding pre-pregnancy underweight and obese women. Sixth, complete-case data analyses were performed by including only participants without missing covariate data. Seventh, we additionally controlled for maternal Mediterranean diet score, calculated based on women’s early-pregnancy dietary intakes (Chatzi et al., 2008). For these analyses, data was restricted to the Rhea cohort, as no information on dietary intake during pregnancy was available for women included in MEFAB. Finally, we assessed the possible mediating effect of delivery mode, birth weight, gestational age, gestational diabetes and children’s BMI on the main associations. The possible mediating effects of child’s blood leptin and tumour necrosis factor $\alpha$ (TNF $\alpha$), measured at 4 years, and cord-blood leptin were assessed in the Rhea cohort as post-hoc analyses.

All statistical analyses were conducted with either Stata version 14.2 (StataCorp, 2015) or R version 3.5.1 (R Core Team, 2008), with α set at 0.05.

## Results

Population characteristics subdivided by cohort are presented in Table 1 (by maternal pre-pregnancy BMI status) and in the Online Resource 4 (by problem behaviour category). In both cohorts, a higher percentage of women with a pre-pregnancy BMI in the overweight/obese range had a low level of education compared to normal-weight women. Furthermore, there was a tendency for children born to women with overweight/obesity to have higher problem behaviour scores compared to children born to women with a normal BMI.

**Table 1:** Population’s characteristic

	MEFAB					Rhea			
	n	Normal weight^a^	Overweight/obese^a^	p-value	n		Normal weight^a^	Overweight/obese^a^	p-value
Maternal characteristics									
Age at delivery (years)	378	29.81 (3.90)	28.90 (4.12)	0.047	413		29.63 (4.50)	30.70 (4.92)	0.026
Ancestry/nationality (% Caucasian/Greek)^b^	377	272 (98.91%)	101 (99.02%)	0.926	410		249 (95.04%)	144 (94.74%)	0.893
Pre-pregnancy BMI (kg/m^2^)^a^	378	21.90 (1.77)	27.28 (25.79, 29.34)	< 0.001	414		21.96 (1.87)	28.40 (26.22, 32.70)	< 0.001
Smoking during pregnancy (% ever smokers)	376	63 (22.99%)	31 (30.39%)	0.141	371		40 (16.74%)	25 (18.94%)	0.593
Alcohol during pregnancy (% ever drinkers)	376	8 (2.92%)	5 (4.90%)	0.350	361		65 (29.02%)	36 (26.28%)	0.574
Parity									
No children	378	200 (72.73%)	81 (78.64%)	0.314	408		127 (49.22%)	58 (38.67%)	0.041
One child		62 (22.55%)	16 (15.53%)				97 (37.60%)	60 (40.00%)	
Two or more children		13 (4.73%)	6 (5.83%)				34 (13.18%)	32 (21.33%)	
Level of education									
Low	263	40 (20.94%)	27 (37.50%)	0.001	414		13 (4.96%)	20 (13.16%)	0.009
Middle		72 (37.70%)	32 (44.44%)				143 (54.58%)	70 (46.05%)	
High		79 (41.36%)	13 (18.06%)				106 (40.46%)	62 (40.79%)	
Weight in pregnancy (kg)									
First trimester	378	61.04 (6.63)	77.52 (11.15)	< 0.001	342		60.77 (6.68)	80.59 (14.06)	< 0.001
Second trimester	377	65.24 (6.63)	80.85 (11.12)	< 0.001	–		–	–	–
Third trimester	376	69.56 (7.16)	84.71 (11.20)	< 0.001	362		70.91 (8.12)	88.68 (13.91)	< 0.001
At delivery	375	72.73 (7.69)	87.88 (11.91)	< 0.001	377		73.24 (8.72)	87.04 (14.48)	< 0.001
Caesarean section (% yes)	376	20 (7.33%)	10 (9.71%)	0.447	413		114 (43.68%)	91 (59.87%)	0.002
Gestational diabetes mellitus (% yes)	376	3 (1.10%)	2 (1.94%)	0.525	379		20 (8.40%)	23 (16.31%)	0.019
Children’s characteristics									
Gestational age (weeks)	378	39.83 (1.53)	39.90 (1.83)	0.680	411		38.28 (1.50)	37.94 (1.73)	0.035
Birth weight (g)	377	3303.64 (509.51)	3305.29 (553.33)	0.978	409		3221.00 (437.39)	3199.67 (480.88)	0.647
Sex (% male)	378	150 (54.55%)	55 (53.40%)	0.842	414		139 (53.05%)	93 (61.18%)	0.108
Breastfeeding (% ever breastfed)	268	98 (50.00%)	27 (37.50%)	0.069	401		223 (88.14%)	121 (81.76%)	0.077
Day-care attendance (% yes)	270	166 (84.69%)	55 (74.32%)	0.049	413		57 (21.84%	27 (17.76%)	0.321
Age at survey (years)	268	7.28 (0.26)	7.27 (0.28)	0.902	413		6.57 (0.28)	6.58 (0.28)	0.616
BMI at survey (kg/m^2^)	264	15.12 (14.31, 16.14)	15.62 (14.59, 17.35)	0.001	412		16.03 (14.99, 17.69)	16.94 (15.73, 19.30)	< 0.001
Total problems	378	50.31 (11.30)	52.82 (10.87)	0.054	414		51.29 (9.75)	52.32 (8.37)	0.275
Internalizing behaviours	378	51.65 (10.78)	52.46 (9.38)	0.506	414		51.26 (9.04)	52.04 (8.66)	0.391
Externalizing behaviours	378	50.28 (10.60)	53.32 (10.52)	0.013	414		53.40 (9.72)	54.61 (8.11)	0.198

^a^Maternal BMI was computed using first-trimester weight in MEFAB and pre-pregnancy BMI in Rhea

^b^Ancestry/nationality was coded as Caucasian/other in MEFAB and Greek/other in Rhea; values are expressed as mean (SD), median (IQR) or number (%) as appropriate; p-values are calculated using Student’s *t*-test for continuous variables or chi square for categorical variables

Mean wGWG was 0.40 (SD = 0.11) kg/week in MEFAB, and 0.41 (SD = 0.05) kg/week in Rhea (p = 0.407). The mean intercept of the linear regression between gestational weight and gestational age was 60.99 kg (SD = 10.71) in MEFAB and 63.26 kg (SD = 12.99) in Rhea (p = 0.008). This value can be compared with the reported pre-pregnancy weight in Rhea (mean = 65.79 kg; SD = 14.21; p < 0.0001).

**Fig. 1:**
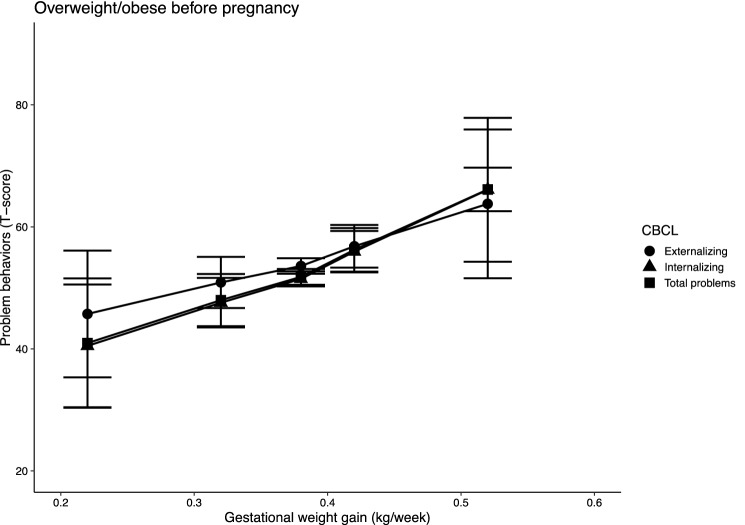
Predicted problem behaviour scores in children of women with pre-pregnancy overweight or obesity. *Note* n = 255; models were adjusted for maternal first trimester (MEFAB) or pre-pregnancy (Rhea) weight, maternal age at delivery, smoking and alcohol consumption during pregnancy, parent’s level of education, parity, children’s sex and children’s age at assessment; 95% confidence intervals are shown

**Fig. 2:**
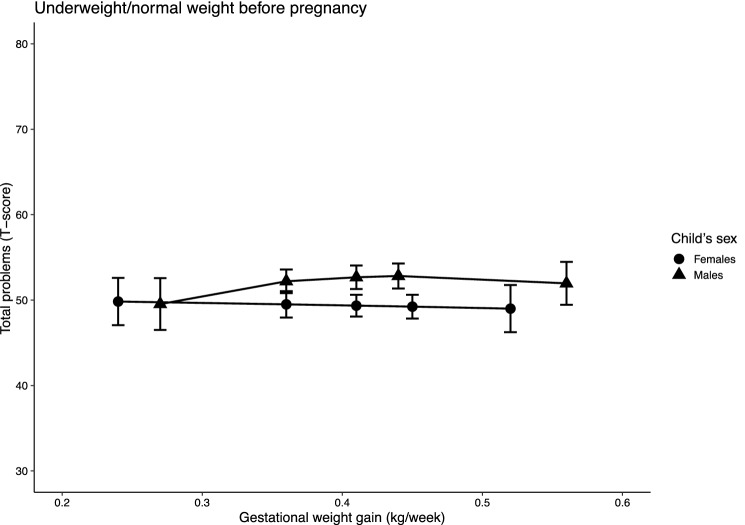
Predicted total problem scores by wGWG in children of women with pre-pregnancy BMI in the underweight or normal ranges, stratified by children’s sex. *Note* n = 289 (males) and 248 (females); models were adjusted for maternal first trimester (MEFAB) or pre-pregnancy (Rhea) weight, maternal age at delivery, smoking and alcohol consumption during pregnancy, parent’s level of education, parity and children’s age at assessment; 95% confidence intervals are shown

**Fig. 3:**
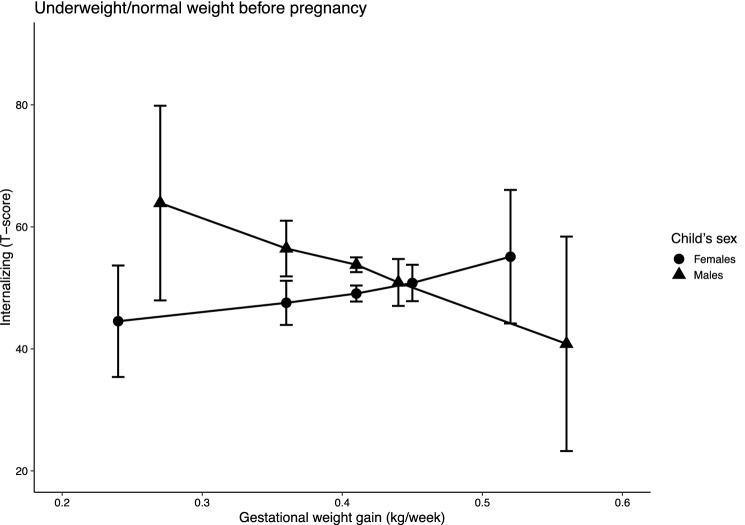
Predicted internalizing behaviour scores by wGWG in children of women with pre-pregnancy BMI in the underweight or normal ranges, stratified by children’s sex. *Note* n = 289 (males) and 248 (females); models were adjusted for maternal first trimester (MEFAB) or pre-pregnancy (Rhea) weight, maternal age at delivery, smoking and alcohol consumption during pregnancy, parent’s level of education, parity and children’s age at assessment; 95% confidence intervals are shown

**Fig. 4:**
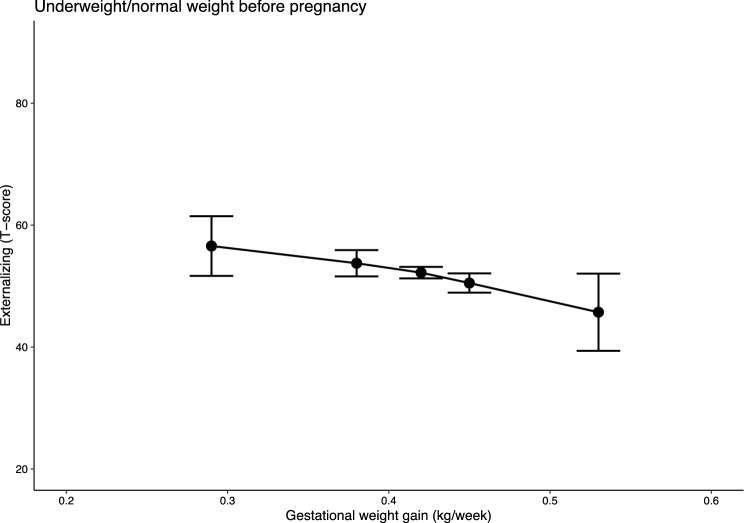
Predicted externalizing behaviour scores by wGWG in children of women with pre-pregnancy BMI in the underweight or normal ranges. *Note* n = 537; models were adjusted for maternal first trimester (MEFAB) or pre-pregnancy (Rhea) weight, maternal age at delivery, smoking and alcohol consumption during pregnancy, parent’s level of education, parity, children’s sex and children’s age at assessment; 95% confidence intervals are shown

The interaction between wGWG and pre-pregnancy BMI was statistically significant on all three outcomes. Furthermore, in the normal pre-pregnancy BMI group statistically significant interactions between wGWG and children’s sex were found on total problems and internalizing behaviours. Besides, interactions between wGWG and children’s sex were not statistically significant on externalizing behaviours in the pre-pregnancy normal weight group and on any outcomes in the pre-pregnancy overweight/obese group. The analyses were, therefore, stratified to account for the two effect modifiers—i.e., pre-pregnancy BMI and children’s sex; results are presented in Figs. 1, 2, 3 and 4, and in the Online Resource 5. Percentiles of wGWG were calculated in each group separately; 5th and 95th percentiles of wGWG corresponded to approximately 0.25 kg/week and 0.55 kg/week, respectively, in all groups (exact estimates are reported in Tables 8 and 9, Online Resource 5). In the pre-pregnancy overweight-obesity group, scores of both total problems and internalizing behaviours were approximately 25 points higher in children born to women who gained the most amount of weight during their pregnancy, compared to children born to women who gained the least weight. Average scores at 5th and 95th percentiles of wGWG (95% confidence interval) were 40.95 (30.35, 51.55) and 66.13 (53.69, 78.57) for total problems, and 40.49 (30.43, 50.54) and 66.08 (54.28, 77.87) for internalizing behaviours. A smaller difference (i.e., 18 points) was found in externalizing behaviour scores (45.73 (35.34, 56.12) and 63.77 (51.58, 75.97), for the 5th and 95th percentiles of wGWG, respectively). It is worth noting that the average predicted problem-behaviour scores for children of women with overweight/obesity who gained about 0.5 kg/week fell within the clinical level of symptomatology (i.e., above 63).

However, in children born to women with a normal pre-pregnancy BMI, internalizing behaviour scores decreased by 23 points for increasing wGWG in males (63.90 (47.95, 79.84) and 40.83 (23.26, 58.41)), while increasing slightly (i.e., about 10 points) in females (44.53 (35.39, 53.67) and 55.11 (44.16, 66.05)). No association was observed in children of women with a normal weight for total problems (males: 49.53 (46.50, 52.56) and 51.96 (49.45, 54.47); females: 49.83 (47.05, 52.60) and 49.00 (46.24, 51.76)), and only a small reduction (i.e., about 10 points) was found in externalizing behaviour scores for increasing wGWG (56.56 (51.66, 61.46) and 45.71 (39.38, 52.03)).

Results of the sensitivity analysis are presented in the Online Resource 6. Overall, with the exception of the association between wGWG and internalizing behaviours in Rhea only, all regressions in the pre-pregnancy overweight/obese group showed similar estimates and a clear increase in the outcome’s predicted score. Besides, the associations between wGWG and problem behaviours in the normal-weight group were inconsistent. Finally, none of the performed mediation analyses highlighted a significant indirect effect, suggesting that the identified variables might not lie in the causal path between wGWG and problem behaviours.

## Discussion

The aim of the present study was to evaluate the association between wGWG and problem behaviours in school-age children by pooling together individual data from two prospective European birth cohorts, MEFAB and Rhea. These results provide evidence for the association between maternal weight in pregnancy and behaviour problems in school-age children. In the overweight/obesity group, we observed a 25-point difference (on a 0–100 scale) in the average scores of the total problem and internalizing behaviour scales between children of women in the lower-end of the wGWG range and children of women in the higher-end of this range. Similarly, externalizing behaviour scores increased by about 18 points in children of women with the highest wGWG. Furthermore, our results showed that the offspring of women who gain excessive weight during pregnancy (i.e., about 0.5 kg/week) may attain mean problem behaviour scores in the clinical range of symptomatology (i.e., over 63). These results are likely to be of clinical relevance, considering that children with behaviour in the clinical range are at an increased risk of poor developmental outcomes, with higher odds for each unit increase in CBCL scores (Ferdinand et al., 2004; Reef et al., 2010).

In contrast, in children of women with a normal pre-pregnancy BMI, the associations were inconsistent. We observed a sex-specific trend of change for increasing wGWG in internalizing behaviours, with scores decreasing in males and slightly increasing in females. A reduction in externalizing behaviours for increasing wGWG was evident in males and females combined, while no association was observed with total problems.

To our knowledge, only one previous study has investigated the association between GWG by maternal pre-pregnancy BMI category and childhood problem behaviours, reporting no statistically significant association (Pugh et al., 2016). However, since the study population comprised only of low-income, high-risk women, these results cannot be directly compared with our findings, which were based on well educated, low-risk families. Other studies examined the association between GWG and infants’ neurobehavior (Aubuchon-Endsley, Bublitz, & Stroud, 2017) and attention deficit/hyperactivity disorder (ADHD) risk (Rodriguez et al., 2008), which are strongly related to problem behaviours in mid-childhood (Biederman, Monuteaux, Kendrick, Klein, & Faraone, 2005; Liu et al., 2010). Poor outcomes are reported in children of obese women who gained excessive weight during pregnancy (Aubuchon-Endsley et al., 2017; Rodriguez et al., 2008), supporting our findings.

If replicated, the results of the present study may have public health relevance, given the constantly rising number of overweight and obese women entering pregnancy (Institute of Medicine and National Research Council, 2009). In line with the American Institute of Medicine guidelines (Institute of Medicine and National Research Council, 2009), we showed that for overweight/obese women an adequate weight gain (i.e., approximately 0.22 kg/week) is associated with the lowest childhood problem behaviours. Given that 50–60% of overweight or obese women gain weight in excess during their pregnancy (Institute of Medicine and National Research Council, 2009), we recommend women with overweight/obesity should be closely monitored to prevent excessive GWG.

Maternal weight in pregnancy might influence the development of children’s behaviour via increased glucose levels and the consequent rise in insulin secretion by the foetus or through elevated levels of inflammatory cytokines. Additionally, obesity might result in leptin resistance, with consequent excessive leptin levels and disproportionate release of cortisol (Edlow, 2017). In fact, a previous study found that evening cortisol levels were elevated in women with pre-pregnancy obesity during the third trimester of pregnancy, with an even greater increase in cortisol levels observed in women with excessive GWG (Aubuchon-Endsley et al., 2014). Despite this evidence, in the present study, gestational diabetes mellitus did not mediate the association between wGWG and problem behaviours; however, only a small number of women in this study were diagnosed with gestational diabetes. Furthermore, we found no evidence of mediation by cord-blood leptin, serum pro-inflammatory cytokine TNF $\alpha$ and leptin in children from the Rhea study. However, no more than 155 children were included in these analyses: the possible mediating effects by gestational diabetes, inflammatory cytokines and leptin cannot be completely ruled out.

Strengths of this study include the pooling of individual data from two European prospective birth cohorts, MEFAB and Rhea, which has led to greater generalisability of the results. Additional strengths include a centralised statistical-analysis approach with harmonised exposure, confounder and outcome variables and the assessment of children’s behaviour using similar versions of the CBCL.

Women’s weight in pregnancy was directly and repeatedly measured by hospital staff in both cohorts, with the exception of weight at delivery in Rhea, which was self-reported. Consequently, we were able to obtain precise estimates of wGWG, comparable between cohorts, by considering the trends of weight gain during pregnancy. Gestational weight trajectories were better described by linear patterns, in contrast with previous publications that showed non-linear GWG (e.g., Fraser et al., 2010). It should be noted, however, that, comparing the intercept of maternal weight’s trajectory with the self-reported pre-pregnancy weight in Rhea, a different pattern of weight gain could be hypothesized for the first weeks of pregnancy, with lower rates of weight gain in this period. Therefore, the frequency of weight measurements during pregnancy might not have been sufficient to capture the full complexity of GWG trajectory. Consequently, the GWG trajectory we described might resemble more the characteristic pattern of the second and third trimesters, overestimating slightly the weight gain in the first trimester.

A few limitations should also be considered. Pre-pregnancy BMI was based on first trimester measured weight in MEFAB and on self-reported pre-pregnancy weight in Rhea. Although not ideal, these methods represent common practice in epidemiological studies and clinical settings, generally being considered reliable and comparable (Headen, Cohen, Mujahid, & Abrams, 2017; Krukowski et al., 2016). In Rhea, delivery weight was self-reported 8–10 weeks postpartum. A systematic review has shown that the recall of delivery weight due to self-report is reproducible and valid (Headen et al., 2017), while underreporting of delivery weight, which tends to be more frequent than over-reporting, would most likely bias estimates toward the null (Schieve et al., 1999). Only few participants were classified as underweight or obese, precluding us from testing our hypotheses in these subgroups. Virtually all women included in this study were Caucasian, therefore these findings cannot be directly extended to other ethnic groups. Although the development of problem behaviours is influenced by several risk factors, we did not assess any child- or family-specific factors exclusively related to infancy or childhood. Nonetheless, these factors cannot be considered potential confounders of the association between GWG and problem behaviours, as they necessarily occur after the exposure (VanderWeele, 2019). Finally, despite their possible association with weight status before pregnancy or wGWG (Hartley, McPhie, Skouteris, Fuller-Tyszkiewicz, & Hill, 2015; Stuebe, Oken, & Gillman, 2009) and children’s development and behaviour (Borge et al., 2017; Madigan et al. 2018), we could not adequately control for maternal psychopathology, diet quality and physical activity before or during pregnancy.

## Conclusions

Increasing wGWG, in combination with pre-pregnancy overweight/obesity, was associated with higher problem behaviours in school-age children. Less clear was the association between wGWG and problem behaviours in children of women with a normal pre-pregnancy BMI. Future studies should further examine the relationship between wGWG and childhood problem behaviours, assessing maternal psychopathology, diet quality and physical activity levels before and during pregnancy and including more women with a pre-pregnancy BMI in the obese or underweight ranges.

## Electronic supplementary material

Below is the link to the electronic supplementary material.

Electronic supplementary material 1 (PDF 127 kb)

Electronic supplementary material 2 (PDF 32 kb)

Electronic supplementary material 3 (PDF 183 kb)

Electronic supplementary material 4 (PDF 200 kb)

Electronic supplementary material 5 (PDF 164 kb)

Electronic supplementary material 6 (PDF 190 kb)

## Abbreviations

CBCL: Child Behaviour Checklist
GWG: Gestational weight gain
wGWG: Weekly gestational weight gain
MEFAB: Maastricht Essential Fatty Acids Birth cohort
