# Supplementary material for: Gestational Weight Gain by Maternal Pre-pregnancy BMI and Childhood Problem Behaviours in School-Age Years: A Pooled Analysis of Two European Birth Cohorts
Source: Matern Child Health J. 2020 Jun 17;24(10):1288–98. doi: 10.1007/s10995-020-02962-y (PMC7476966; doi:10.1007/s10995-020-02962-y)
Supplement: Supplementary file 1 — Electronic supplementary material 1 (PDF 127 kb) [file 10995_2020_2962_MOESM1_ESM.pdf]

Electronic Supplementary Material

Gestational weight gain by maternal pre-pregnancy BMI and childhood problem behaviours in school-age years: a pooled analysis of two European birth cohorts

Maternal and Child Health Journal

Non-response analyses

Table 2A: Included and excluded participants' characteristics, MEFAB

	Included		Excluded		
	n	n (%) or mean (SD) or median (IQR)	n	n (%) or mean (SD) or median (IQR)	p-value
**Maternal characteristics**					
Age at delivery (years)	378	29.56 (3.97)	901	29.18 (4.42)	0.148
Ancestry (% Caucasian)	377	373 (98.94%)	884	860 (97.29%)	0.068
Pre-pregnancy BMI (kg/m2)	378	22.90 (21.49, 25.23)	810	23.46 (21.22, 26.30)	0.030
Pre-pregnancy BMI (% Overweight/obese)	378	103 (27.25%)	810	277 (34.20%)	0.017
Smoking during pregnancy (% ever smokers)	376	94 (25.00%)	884	234 (26.47%)	0.586
Alcohol during pregnancy (% ever drinkers)	376	13 (3.46%)	884	22 (2.49%)	0.338
Parity	378		894		0.985
First born		281 (74.34%)		664 (74.27%)	
Second born		78 (20.63%)		183 (20.47%)	
Third + born		19 (5.03%)		47 (5.26%)	
Level of education	263		33		0.012
Low		67 (25.48%)		4 (12.12%)	
Middle		104 (39.54%)		22 (66.67%)	
High		92 (34.98%)		7 (21.21%)	
Weight in pregnancy (kg)					
First trimester	378	65.53 (10.94)	868	66.85 (12.59)	0.077
Second trimester	377	69.47 (10.79)	872	70.97 (12.58)	0.043
Third trimester	376	73.67 (10.80)	863	75.48 (12.82)	0.017
At delivery	375	76.89 (11.28)	873	78.55 (13.14)	0.033
Caesarean section (% yes)	376	30 (7.98%)	891	123 (13.80%)	0.004
Gestational diabetes mellitus (% yes)	376	5 (1.33%)	881	30 (3.41%)	0.041
**Children's characteristics**					
Gestational age (weeks)	378	39.85 (1.61)	828	39.81 (2.56)	0.807
Birth weight (g)	377	3304.09 (520.98)	892	3205.14 (583.27)	0.005
Sex (% male)	378	205 (54.23%)	896	492 (54.91%)	0.824
Breastfeeding (% ever breastfed)	268	125 (46.64%)	20	9 (45.00%)	0.887

Table 2B: Included and excluded participants' characteristics, Rhea

	Included		Excluded		
	**n**	**n (%) or mean (SD) or median (IQR)**	**n**	**n (%) or mean (SD) or median (IQR)**	**p-value**
**Maternal characteristics**					
Age at delivery (years)	413	30.02 (4.68)	1057	29.10 (5.22)	0.002
Nationality (% Greek)	410	393 (95.85%)	1071	956 (89.26%)	<0.001
Pre-pregnancy BMI (kg/m2)	414	23.61 (21.67, 26.72)	959	23.18 (20.81, 26.13)	0.006
Pre-pregnancy BMI (% Overweight/obese)	414	152 (36.71%)	959	312 (32.53%)	0.133
Smoking during pregnancy (% ever smokers)	371	65 (17.52%)	869	198 (22.78%)	0.038
Alcohol during pregnancy (% ever drinkers)	361	101 (27.98%)	759	216 (28.46%)	0.867
Parity	408		990		0.123
First born		185 (45.34%)		412 (41.62%)	
Second born		157 (38.48%)		372 (37.58%)	
Third + born		66 (16.18%)		206 (20.81%)	
Level of education	414		991		<0.001
Low		33 (7.97%)		181 (18.26%)	
Middle		213 (51.45%)		505 (50.96%)	
High		168 (40.58%)		305 (30.78%)	
Weight in pregnancy (kg)					
First trimester	342	67.49 (13.58)	793	67.11 (13.90)	0.672
Second trimester	-	-	-	-	-
Third trimester	362	77.58 (13.70)	817	75.80 (12.71)	0.031
At delivery	377	78.29 (13.00)	929	76.63 (12.82)	0.035
Caesarean section (% yes)	413	205 (49.64%)	1076	561 (52.14%)	0.387
Gestational diabetes mellitus (% yes)	379	43 (11.35%)	828	101 (12.20%)	0.672
**Children's characteristics**					
Gestational age (weeks)	411	38.16 (1.59)	1063	38.12 (1.77)	0.672
Birth weight (g)	409	3213.18 (453.36)	1015	3121.06 (488.82)	0.001
Sex (% male)	414	232 (56.04%)	1108	535 (48.29%)	0.007
Breastfeeding (% ever breastfed)	401	344 (85.79%)	891	746 (83.73%)	0.346
