# Supplementary material for: Gestational Weight Gain by Maternal Pre-pregnancy BMI and Childhood Problem Behaviours in School-Age Years: A Pooled Analysis of Two European Birth Cohorts
Source: Matern Child Health J. 2020 Jun 17;24(10):1288–98. doi: 10.1007/s10995-020-02962-y (PMC7476966; doi:10.1007/s10995-020-02962-y)
Supplement: Supplementary file 2 — Electronic supplementary material 2 (PDF 32 kb) [file 10995_2020_2962_MOESM2_ESM.pdf]

Electronic Supplementary Material

Gestational weight gain by maternal pre-pregnancy BMI and childhood problem behaviours in school-age years: a pooled analysis of two European birth cohorts

Maternal and Child Health Journal

Additional data regarding the computation of GWG trajectories

In the first step of the analyses, to improve the modelling precision, all women with available weight data during pregnancy were included. This resulted in a total of 1227 women in MEFAB and 1353 women in Rhea.
An overview of the available data is provided below.

Table 3a: Participation rate during pregnancy in MEFAB, by measurement session

Measurement session	Weight measurements (n,%)
First (week 10)	1213 (98.86%)
Second (week 21)	1215 (99.02%)
Third (week 32)	1204 (98.13%)
Fourth (delivery)	1215 (99.02%)

Table 3b: Participation rate during pregnancy in Rhea, by measurement session

Measurement session	Weight measurements (n,%)
First (week 12)	1127 (83.29%)
Second (week 32)	1102 (81.45%)
Third (delivery)	1220 (90.17%)

The median (IQR) number of measurements per woman was 4 (4, 4) in MEFAB and 3 (2, 3) in Rhea. The percentages of women with one weight measurement were 0.24% (MEFAB) and 6.95% (Rhea).

Predicted weight were calculated in an intermediate step of the analysis, before the computation of wGWG. We checked the validity of our prediction by calculating the correlation between measured and predicted weight values ($r = 0.997$ in MEFAB and $r=0.98$ in Rhea; $p<0.0001$ in both cases).
