# Supplementary material for: Gestational Weight Gain by Maternal Pre-pregnancy BMI and Childhood Problem Behaviours in School-Age Years: A Pooled Analysis of Two European Birth Cohorts
Source: Matern Child Health J. 2020 Jun 17;24(10):1288–98. doi: 10.1007/s10995-020-02962-y (PMC7476966; doi:10.1007/s10995-020-02962-y)
Supplement: Supplementary file 3 — Electronic supplementary material 3 (PDF 183 kb) [file 10995_2020_2962_MOESM3_ESM.pdf]

Electronic Supplementary Material

Gestational weight gain by maternal pre-pregnancy BMI and childhood problem behaviours in school-age years: a pooled analysis of two European birth cohorts

Maternal and Child Health Journal

Directed acyclic graph used for variable selection

Figure 3: Directed acyclic graph (DAG) used for variable selection

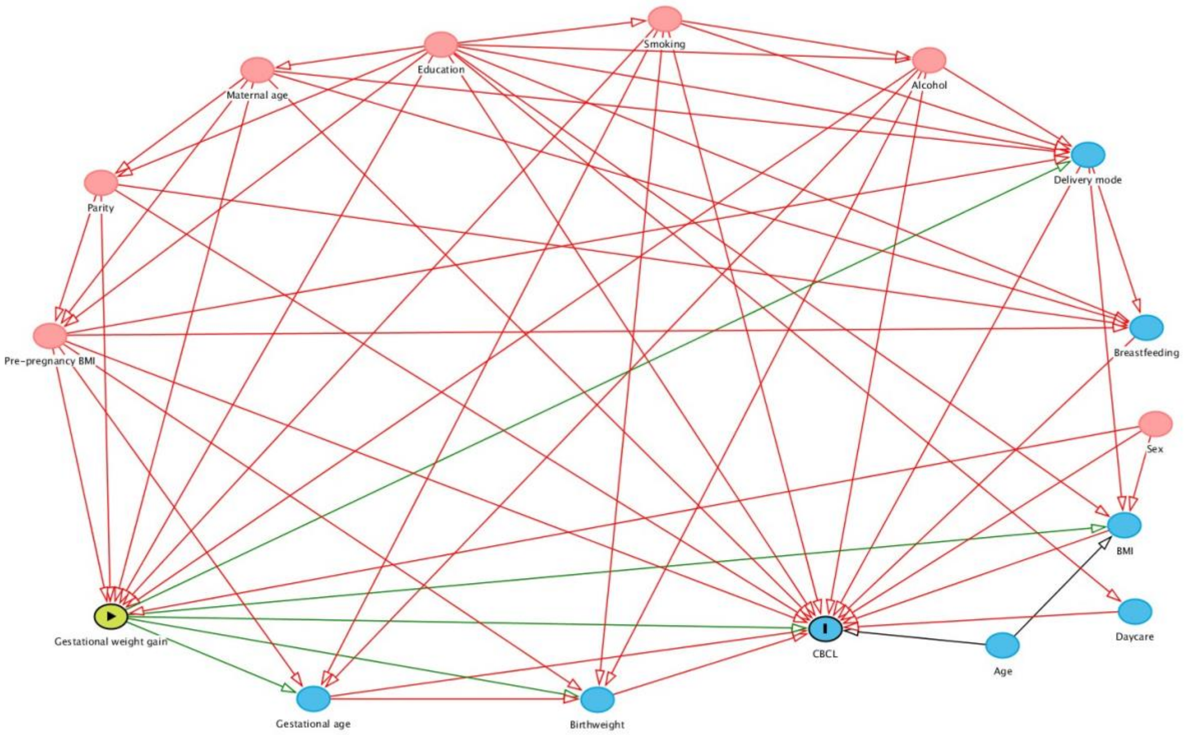

Note: Green arrows represent causal paths from the exposure; red arrows represent biasing paths to the exposure and/or the outcome. Red ovals represent the minimal sufficient adjustment set for estimating the total effect of wGWG on childhood problem behaviours. Maternal variables: Gestational weight gain (*i.e.,* wGWG), pre-pregnancy BMI, parity, maternal age, smoking, alcohol, education (combined maternal and paternal), delivery mode. Children's variables: CBCL (*i.e.,* problem behaviours), sex, age, gestational age, birthweight, breastfeeding, BMI, day-care.
