# Supplementary material for: Gestational Weight Gain by Maternal Pre-pregnancy BMI and Childhood Problem Behaviours in School-Age Years: A Pooled Analysis of Two European Birth Cohorts
Source: Matern Child Health J. 2020 Jun 17;24(10):1288–98. doi: 10.1007/s10995-020-02962-y (PMC7476966; doi:10.1007/s10995-020-02962-y)
Supplement: Supplementary file 4 — Electronic supplementary material 4 (PDF 200 kb) [file 10995_2020_2962_MOESM4_ESM.pdf]

Electronic Supplementary Material

Gestational weight gain by maternal pre-pregnancy BMI and childhood problem behaviours in school-age years: a pooled analysis of two European birth cohorts

Maternal and Child Health Journal

Population characteristics by category of problem behaviour scales
Problem behaviour scales were categorised into normal development (score lower than 63) and clinical range (score over or equal to 63).

Table 4A: Population's characteristics subdivided by category of total problems

	MEFAB			Rhea		
	**Normal development (n=310)**	**Clinical development (n=68)**	**p-value**	**Normal development (n=368)**	**Clinical development (n=46)**	**p-value**
**Maternal characteristics**						
Age at delivery (years)	29.91 (3.91)	27.96 (3.88)	<0.001	30.14 (4.64)	29.11 (4.93)	0.161
Ancestry/Nationality (% Caucasian/Greek)[b]	305 (98.71%)	68 (100%)	0.346	350 (95.11%)	43 (93.48%)	0.635
Pre-pregnancy BMI (kg/m2)[a]	23.51 (3.49)	24.31 (4.59)	0.105	24.88 (5.16)	25.32 (4.79)	0.577
Smoking during pregnancy (% ever smokers)	66 (21.43%)	28 (41.18%)	0.001	57 (17.38%)	8 (18.60%)	0.842
Alcohol during pregnancy (% ever drinkers)	11 (3.57%)	2 (2.94%)	0.797	88 (27.33%)	13 (33.33%)	0.430
Parity			0.165			0.583
No children	225 (72.58%)	56 (82.35%)		163 (45.03%)	22 (47.83%)	
One child	67 (21.61%)	11 (16.18%)		138 (38.12%)	19 (41.30%)	
Two or more children	18 (5.81%)	1 (1.47%)		61 (16.85%)	5 (10.87%)	
Level of education			0.024			0.038
Low	48 (22.12%)	19 (41.30%)		27 (7.34%)	6 (13.04%)	
Middle	89 (41.01%)	15 (32.61%)		184 (50.00%)	29 (63.04%)	
High	80 (36.87%)	12 (26.09%)		157 (42.66%)	11 (23.91%)	
Weight in pregnancy (kg)						
First trimester	64.98 (10.33)	68.01 (13.16)	0.039	66.99 (13.36)	71.74 (14.90)	0.047
Second trimester	68.94 (10.23)	71.86 (12.86)	0.044	-	-	-
Third trimester	73.23 (10.36)	75.69 (12.51)	0.092	77.22 (13.63)	80.39 (14.12)	0.064
At delivery	76.29 (10.70)	79.61 (13.35)	0.028	77.75 (12.76)	82.95 (14.25)	0.018
Caesarean section (% yes)	24 (7.79%)	6 (8.82%)	0.776	181 (49.32%)	24 (52.17%)	0.715
Gestational diabetes mellitus (% yes)	4 (1.29%)	0 (0.00%)	0.346	4 (1.10%)	4 (8.89%)	<0.001

Table 4A (continued)

	MEFAB			Rhea		
	Normal development (n=310)	Clinical development (n=68)	p-value	Normal development (n=368)	Clinical development (n=46)	p-value
**Children's characteristics**						
Gestational age (weeks)	39.84 (1.61)	39.89 (1.63)	0.823	38.17 (1.54)	38.02 (1.99)	0.539
Birth weight (g)	3308.17 (518.62)	3285.52 (535.07)	0.746	3223.83 (448.63)	3129.13 (486.10)	0.182
Sex (% male)	165 (53.23%)	40 (58.82%)	0.401	200 (54.35%)	32 (69.57%)	0.050
Breastfeeding (% ever breastfed)	103 (46.82%)	22 (45.83%)	0.901	308 (86.03%)	36 (83.72%)	0.682
Day-care attendance (% yes)	187 (83.48%)	34 (73.91%)	0.125	71 (19.29%)	13 (28.89%)	0.131
Age at survey (years)	7.28 (0.25)	7.28 (0.30)	0.947	6.56 (0.28)	6.62 (0.29)	0.240
BMI at survey (kg/m^2)	15.60 (1.83)	15.78 (1.77)	0.533	16.91 (2.64)	18.06 (3.11)	0.007

Table 4B: Population’s characteristics subdivided by category of internalizing behaviours

	MEFAB			Rhea		
	**Normal development (n=321)**	**Clinical development (n=57)**	**p-value**	**Normal development (n=365)**	**Clinical development (n=49)**	**p-value**
**Maternal characteristics**						
Age at delivery (years)	29.71 (3.97)	28.69 (3.89)	0.074	30.09 (4.61)	29.50 (5.23)	0.412
Ancestry/Nationality (% Caucasian/Greek)[b]	316 (98.75%)	57 (100%)	0.396	345 (94.52%)	48 (97.96%)	0.303
Pre-pregnancy BMI (kg/m^2)[a]	23.74 (3.80)	23.17 (3.24)	0.289	24.93 (5.27)	24.93 (3.83)	0.997
Smoking during pregnancy (% ever smokers)	75 (23.51%)	19 (33.33%)	0.115	58 (17.79%)	7 (15.56%)	0.771
Alcohol during pregnancy (% ever drinkers)	11 (3.45%)	2 (3.51%)	0.982	88 (27.59%)	13 (33.95%)	0.648
Parity			0.432			0.713
No children	236 (73.52%)	45 (78.95%)		162 (45.00%)	23 (47.92%)	
One child	67 (20.87%)	11 (19.30%)		141 (39.17%)	16 (33.33%)	
Two or more children	18 (5.61%)	1 (1.75%)		57 (15.83%)	9 (18.75%)	
Level of education			0.451			0.102
Low	54 (24.11%)	13 (33.33%)		28 (7.67%)	5 (10.20%)	
Middle	91 (40.62%)	13 (33.33%)		182 (49.86%)	31 (63.27%)	
High	79 (35.27%)	13 (33.33%)		155 (42.47%)	13 (26.53%)	
Weight in pregnancy (kg)						
First trimester	65.60 (11.10)	65.10 (10.02)	0.749	67.28 (13.67)	69.09 (12.93)	0.429
Second trimester	69.50 (10.88)	69.27 (10.41)	0.880	-	-	-
Third trimester	73.72 (10.81)	73.37 (10.83)	0.475	77.65 (13.88)	77.13 (12.53)	0.814
At delivery	76.87 (11.29)	76.98 (11.36)	0.947	78.12 (12.80)	79.63 (14.56)	0.475
Caesarean section (% yes)	27 (8.46%)	3 (5.26%)	0.411	180 (49.45%)	25 (51.02%)	0.837
Gestational diabetes mellitus (% yes)	4 (1.25%)	0 (0.00%)	0.397	6 (1.68%)	2 (4.08%)	0.255

Table 4B (continued)

	MEFAB			Rhea		
	**Normal development (n=321)**	**Clinical development (n=57)**	**p-value**	**Normal development (n=365)**	**Clinical development (n=49)**	**p-value**
**Children's characteristics**						
Gestational age (weeks)	39.81 (1.66)	40.08 (1.33)	0.242	38.17 (1.56)	38.10 (1.84)	0.803
Birth weight (g)	3289.25 (527.00)	3387.37 (481.69)	0.191	3204.42 (440.49)	3277.55 (539.48)	0.290
Sex (% male)	175 (54.52%)	30 (52.63%)	0.792	190 (52.05%)	42 (85.71%)	<0.001
Breastfeeding (% ever breastfed)	108 (47.37%)	17 (42.50%)	0.569	303 (85.59%)	41 (87.23%)	0.762
Day-care attendance (% yes)	189 (82.17%)	32 (80.00%)	0.742	72 (19.73%)	12 (25.00%)	0.393
Age at survey (years)	7.27 (0.25)	7.34 (0.30)	0.129	6.56 (0.28)	6.64 (0.30)	0.061
BMI at survey (kg/m^2)	15.56 (1.74)	16.03 (2.20)	0.106	16.91 (2.48)	17.98 (4.00)	0.010

Table 4C: Population's characteristics subdivided by category of externalizing behaviours

	MEFAB			Rhea		
	**Normal development (n=325)**	**Clinical development (n=53)**	**p-value**	**Normal development (n=357)**	**Clinical development (n=57)**	**p-value**
**Maternal characteristics**						
Age at delivery (years)	29.78 (3.91)	28.19 (4.11)	0.007	30.19 (4.71)	28.98 (4.35)	0.071
Ancestry/Nationality (% Caucasian/Greek)[b]	320 (98.77%)	53 (100%)	0.416	340 (95.24%)	53 (92.98%)	0.471
Pre-pregnancy BMI (kg/m^2)[a]	23.50 (3.50)	24.56 (4.80)	0.054	24.95 (5.11)	24.77 (5.19)	0.798
Smoking during pregnancy (% ever smokers)	75 (23.22%)	19 (35.85%)	0.049	53 (16.61%)	12 (23.08%)	0.256
Alcohol during pregnancy (% ever drinkers)	12 (3.72%)	1 (1.89%)	0.500	83 (26.27%)	18 (40.00%)	0.055
Parity			0.512			0.396
No children	241 (74.15%)	40 (75.47%)		158 (45.01%)	27 (47.37%)	
One child	66 (20.31%)	12 (22.64%)		139 (39.60%)	18 (31.58%)	
Two or more children	18 (5.54%)	1 (1.89%)		54 (15.38%)	12 (21.05%)	
Level of education			0.058			0.327
Low	54 (23.18%)	13 (43.33%)		28 (7.84%)	5 (8.77%)	
Middle	95 (40.77%)	9 (30.00%)		179 (50.14%)	34 (59.65%)	
High	84 (36.05%)	8 (26.67%)		150 (42.02%)	18 (31.58%)	
Weight in pregnancy (kg)						
First trimester	65.04 (10.36)	68.55 (13.68)	0.030	67.13 (13.32)	69.86 (15.12)	0.210
Second trimester	69.03 (10.30)	72.16 (13.23)	0.050	-	-	-
Third trimester	73.34 (10.44)	75.71 (12.76)	0.143	77.37 (13.68)	78.91 (13.88)	0.461
At delivery	76.47 (10.78)	79.45 (13.82)	0.075	77.91 (12.93)	80.71 (13.26)	0.154
Caesarean section (% yes)	26 (8.05%)	4 (7.55%)	0.900	178 (50.00%)	27 (47.37%)	0.712
Gestational diabetes mellitus (% yes)	4 (1.23%)	0 (0.00%)	0.417	6 (1.70%)	2 (3.64%)	0.337

Table 4C (continued)

	MEFAB			Rhea		
	**Normal development (n=325)**	**Clinical development (n=53)**	**p-value**	**Normal development (n=357)**	**Clinical development (n=57)**	**p-value**
**Children's characteristics**						
Gestational age (weeks)	39.83 (1.62)	39.96 (1.58)	0.593	38.12 (1.58)	38.40 (1.70)	0.211
Birth weight (g)	3300.88 (504.59)	3323.68 (616.90)	0.768	3214.32 (449.56)	3205.98 (480.78)	0.898
Sex (% male)	176 (54.15%)	29 (54.72%)	0.939	193 (54.06%)	39 (68.42%)	0.043
Breastfeeding (% ever breastfed)	124 (52.54%)	19 (59.38%)	0.467	298 (85.88%)	46 (85.19%)	0.892
Day-care attendance (% yes)	200 (83.33%)	21 (70.00%)	0.074	66 (18.54%)	18 (31.58%)	0.023
Age at survey (years)	7.27 (0.26)	7.32 (0.30)	0.337	6.56 (0.28)	6.62 (0.32)	0.135
BMI at survey (kg/m_2)	15.62 (1.86)	15.67 (1.56)	0.889	17.07 (2.79)	16.83 (2.18)	0.525
