# Supplementary material for: Gestational Weight Gain by Maternal Pre-pregnancy BMI and Childhood Problem Behaviours in School-Age Years: A Pooled Analysis of Two European Birth Cohorts
Source: Matern Child Health J. 2020 Jun 17;24(10):1288–98. doi: 10.1007/s10995-020-02962-y (PMC7476966; doi:10.1007/s10995-020-02962-y)
Supplement: Supplementary file 5 — Electronic supplementary material 5 (PDF 164 kb) [file 10995_2020_2962_MOESM5_ESM.pdf]

Electronic Supplementary Material

Gestational weight gain by maternal pre-pregnancy BMI and childhood problem behaviours in school-age years: a pooled analysis of two European birth cohorts

Maternal and Child Health Journal

Additional information about analyses' results

Identification of the best-fitting fractional polynomials of wGWG

The best-fitting fractional polynomials of wGWG were identified separately in category of maternal pre-pregnancy BMI and by child's sex (in case of a statistically significant interaction between sex and wGWG).
We used the "FRACPOLY" command in Stata 14 (StataCorp, 2015) to run a series of models in which wGWG was raised to several combinations of powers (each of the following single powers, plus each combination of two powers: −2, −1, −0.5, 0, 0.5, 1, 2, 3, where a power of zero is the log function), resulting in a wide range of possible curves (Long & Ryoo, 2010). For each group, the best-fitting fractional polynomial model was the one with the lowest deviance.

Details of the best-fitting fractional polynomial model for each group are provided below. In the present analyses, all identified models included a combination of two powers. That is, wGWG was always included in the final models twice, each time with a different power.

Associations between gestational weight gain and problem behaviour scales in children of overweight/obese women (pooled analysis)

The best-fitting fractional polynomial models for the association between gestational weight gain (wGWG) and problem behaviours had the following form:

Total problems = $\beta_0 + \beta_1 * wGWG_3 + \beta_2 * wGWG_3 * \ln(wGWG) + \varepsilon$
Internalizing = $\beta_0 + \beta_1 * wGWG_3 + \beta_2 * wGWG_3 * \ln(wGWG) + \varepsilon$
Externalizing = $\beta_0 + \beta_1 * wGWG_3 + \beta_2 * wGWG_3 * \ln(wGWG) + \varepsilon$

were β_0 denotes the intercept, β_1 and β_2 denote the slopes and ε denotes the error term. Slope estimates are presented in the table 3 below.

Table 5: Estimates for the associations between gestational weight gain and problem behaviour scales in children of overweight/obese women.

	**Total problems**	**Internalizing behaviours**	**Externalizing behaviours**
	**β (95% C.I.)**	**β (95% C.I.)**	**β (95% C.I.)**
β1	90.60* (6.98, 174.21)	95.18* (15.91, 174.45)	60.15 (-21.80, 142.11)
β2	180.16* (14.08, 346.25)	177.69* (20.09, 335.30)	137.09 (-25.77, 299.95)

Note: n=255; all models were adjusted for maternal first trimester (MEFAB) or pre-pregnancy (Rhea) weight, maternal age at delivery, smoking and alcohol consumption during pregnancy, parent’s level of education, parity, and children’s sex, age at assessment and cohort.
*: $p \leq 0.05$

Associations between gestational weight gain and problem behaviours in children of normal-weight women (pooled analysis)

The best-fitting fractional polynomial models for the association between gestational weight gain (wGWG) and problem behaviours had the following form:

Male children:

Total problems = $\beta_0 + \beta_1 * wGWG + \beta_2 * wGWG^2 + \varepsilon$

Internalizing = $\beta_0 + \beta_1 * \ln(wGWG) + \beta_2 * \ln(wGWG) * \ln(wGWG) + \varepsilon$

Female children:

Total problems = $\beta_0 + \beta_1 * wGWG^{-2} + \beta_2 * wGWG^2 + \varepsilon$

Internalizing = $\beta_0 + \beta_1 * wGWG^3 + \beta_2 * wGWG^3 * \ln(wGWG) + \varepsilon$

Externalizing = $\beta_0 + \beta_1 * wGWG^2 + \beta_2 * wGWG^2 * \ln(wGWG) + \varepsilon$

were β_0 denotes the intercept, β_1 and β_2 denote the slopes and ε denotes the error term. Slope estimates are presented in tables 4 and 5 below.

Table 6: Estimates for the associations between gestational weight gain and problem behaviour scales in children of normal weight women, stratified by children's sex.

	**Total problems**	**Internalizing behaviours**
	**β (95% C.I.)**	**β (95% C.I.)**
**Males**		
β_1	102.42* (2.74, 212.10)	-13.33 (-42.89, 12.23)
β_2	-1117.43§ (-236.38, 1.51)	-11.48 (-25.42, 2.46)
**Females**		
β_1	0.02 (-0.07, 0.10)	27.07 (-41.56, 95.70)
β_2	-4.72 (-26.03, 16.59)	99.15 (-61.43, 259.74)

Note: n= 289 (males) and 248 (females); all models were adjusted for maternal first trimester (MEFAB) or pre-pregnancy (Rhea) weight, maternal age at delivery, smoking and alcohol consumption during pregnancy, parent's level of education, parity, children's age at assessment and cohort.

§: $p \leq 0.06$; *: $p \leq 0.05$

For the externalizing scale, the interaction of child’s sex with wGWG was not statistically significant, therefore estimates are presented for males and females combined (table 5).

Table 7: Estimates for the associations between gestational weight gain and externalizing behaviours in children of normal weight women

	**Externalizing behaviours**
	**β (95% C.I.)**
β_1	-27.97*<br>(-54.89, -1.04)
β_2	-67.51<br>(-145.83, 10.80)

Note: n= 537; all models were adjusted for maternal first trimester (MEFAB) or pre-pregnancy (Rhea) weight, maternal age at delivery, smoking and alcohol consumption during pregnancy, parent’s level of education, parity, children’s sex, children’s age at assessment and cohort.

*: $p \leq 0.05$

Problem behaviour score predictions by percentiles of weekly gestational weight gain

Table 8: Total problem and internalizing behaviour scores' predictions by percentiles of wGWG and maternal pre-pregnancy BMI category.

**Weekly gestational weight gain**			**Total problems**	**Internalizing behaviours**
			**Normal BMI**	
**Percentiles**	**Kg/week**	**Children's sex**	**Prediction (95% C.I.)**	
5th	0.27	males	49.53 (46.50, 52.56)	63.90 (47.95, 79.84)
	0.24	females	49.83 (47.05, 52.60)	44.53 (35.39, 53.67)
25th	0.36	males	52.20 (50.81, 53.58)	56.45 (51.89, 61.01)
	0.36	females	49.50 (47.96, 51.05)	47.55 (43.93, 51.18)
50th	0.41	males	52.67 (51.29, 54.05)	53.79 (52.59, 55.00)
	0.41	females	49.35 (48.07, 50.63)	49.07 (47.75, 50.38)
75th	0.44	males	52.83 (51.37, 54.30)	50.89 (47.05, 54.73)
	0.45	females	49.23 (47.84, 50.63)	50.81 (47.83, 53.80)
95th	0.56	males	51.96 (49.45, 54.47)	40.83 (23.26, 58.41)
	0.52	females	49.00 (46.24, 51.76)	55.11 (44.16, 66.05)
			**Overweight/Obese**	
**Percentiles**	**Kg/week**		**Prediction (95% C.I.)**	
5th	0.22		40.95 (30.35, 51.55)	40.49 (30.43, 50.54)
25th	0.32		47.99 (43.71, 52.26)	47.57 (43.51, 51.63)
50th	0.38		51.80 (50.51, 53.10)	51.47 (50.24, 52.70)
75th	0.42		56.24 (52.67, 59.81)	55.94 (52.55, 59.33)
95th	0.52		66.13 (53.69, 78.57)	66.08 (54.28, 77.87)

Note: all models were adjusted for maternal first trimester (MEFAB) or pre-pregnancy (Rhea) weight, maternal age at delivery, smoking and alcohol consumption during pregnancy, parent's level of education, parity, children's age at assessment and cohort. Children's sex was additionally controlled for in non-stratified models.

Table 9: Externalizing behaviour scores' predictions by percentiles of wGWG and maternal pre-pregnancy BMI category.

**Weekly gestational weight gain**		**Externalizing behaviours**
		**Normal BMI**
**Percentiles**	**Kg/week**	**Prediction (95% C.I.)**
5th	0.29	56.56 (51.66, 61.46)
25th	0.38	53.74 (51.58, 55.89)
50th	0.42	52.20 (51.25, 53.15)
75th	0.45	50.49 (48.91, 52.08)
95th	0.53	45.71 (39.38, 52.03)
		**Overweight/Obese**
**Percentiles**	**Kg/week**	**Prediction (95% C.I.)**
5th	0.22	45.73 (35.34, 56.12)
25th	0.32	50.89 (46.70, 55.08)
50th	0.38	53.58 (52.31, 54.85)
75th	0.42	56.82 (53.32, 60.32)
95th	0.52	63.77 (51.58, 75.97)

Note: all models were adjusted for maternal first trimester (MEFAB) or pre-pregnancy (Rhea) weight, maternal age at delivery, smoking and alcohol consumption during pregnancy, parent's level of education, parity, children's sex, children's age at assessment and cohort.

References:

Long, J., & Ryoo, J. (2010). Using fractional polynomials to model non-linear trends in longitudinal data. *British Journal of Mathematical and Statistical Psychology*, *63*(1), 177–203.

StataCorp. (2015). Stata Statistical Software: Release 14. College Station, TX: StataCorp LP.
