# Supplementary material for: Gestational Weight Gain by Maternal Pre-pregnancy BMI and Childhood Problem Behaviours in School-Age Years: A Pooled Analysis of Two European Birth Cohorts
Source: Matern Child Health J. 2020 Jun 17;24(10):1288–98. doi: 10.1007/s10995-020-02962-y (PMC7476966; doi:10.1007/s10995-020-02962-y)
Supplement: Supplementary file 6 — Electronic supplementary material 6 (PDF 190 kb) [file 10995_2020_2962_MOESM6_ESM.pdf]

Electronic Supplementary Material

Gestational weight gain by maternal pre-pregnancy BMI and childhood problem behaviours in school-age years: a pooled analysis of two European birth cohorts

Maternal and Child Health Journal

Sensitivity analyses

Table 10: Results of sensitivity analyses (outcome: total problems)

Sensitivity analyses	Stratification group	wGWG estimates		Predicted scores (SE) range[a]
1. Gestational age >36 complete weeks	Normal BMI, males	80.55	-94.45	51.15 (1.72)
		(-41.11, 202.21)	(-230.04, 41.14)	51.09 (1.52)
	Normal BMI, females	0.02	-2.70	49.79 (1.44)
		(-0.07, 0.10)	(-24.37, 18.96)	49.41 (1.44)
	Overweight	112.87*	210.53*	38.61 (5.66)
		(25.00, 200.74)	(35.43, 385.62)	69.00 (6.66)
2. Complete gestational weight data	Normal BMI, males	102.26§	-108.00	49.01 (1.60)
		(-3.54, 208.06)	(-228.25, 12.25)	51.91 (1.54)
	Normal BMI, females	0.01	-5.95	50.19 (1.50)
		(-0.07, 0.09)	(-28.07, 16.16)	49.03 (1.48)
	Overweight	95.60*	187.66*	39.78 (5.89)
		(9.01, 182.20)	(13.77, 361.56)	67.51 (6.89)
3. Analyses by cohort	MEFAB, Normal BMI, males	132.30*	-141.05*	47.12 (1.87)
		(19.27, 245.32)	(-269.23, -12.87)	50.80 (1.94)
	MEFAB, Normal BMI, females	0.01	-5.27	50.42 (1.90)
		(-0.08, 0.11)	(-31.68, 21.13)	49.26 (2.19)
	MEFAB, Overweight/Obese	98.07	196.18	36.06 (9.32)
		(-9.20, 205.34)	(-24.83, 417.20)	74.04 (11.71)
	Rhea, Normal BMI, males	-86.64	68.84	55.47 (1.99)
		(-525.09, 351.81)	(-428.11, 565.78)	51.60 (1.97)
	Rhea, Normal BMI, females	-0.84	-14.12	50.69 (10.17)
		(-7.47, 5.80)	(-231.92, 203.68)	46.40 (12.30)
	Rhea, Overweight/Obese	39.51	114.07	48.64 (13.60)
		(-375.21, 454.22)	(-523.72, 751.85)	56.49 (15.17)
4. Additional control for breastfeeding and day-care attendance	Normal BMI, males	103.62*	-114.86§	49.77 (1.53)
		(-0.14, 207.37)	(-232.76, 3.04)	51.78 (1.27)
	Normal BMI, females	0.02	-6.51	50.02 (1.42)
		(-0.07, 0.10)	(-28.15, 15.14)	48.82 (1.42)
	Overweight/obese	90.81*	183.11*	40.82 (5.39)
		(7.00, 174.62)	(16.50, 349.72)	66.26 (6.33)
5. Analyses excluding underweight and obese women	Normal BMI, males	99.96	-107.62	49.69 (1.50)
		(-9.87, 209.79)	(-234.00, 18.75)	52.33 (1.26)
	Normal BMI, females	0.01	-3.77	49.68 (1.46)
		(-0.07, 0.10)	(-25.57, 18.03)	49.02 (1.44)
	Overweight	112.30*	280.10**	35.93 (6.39)
		(8.13, 216.48)	(75.50, 484.70)	70.24 (7.56)
6. Complete-case analyses	Normal BMI, males	93.37	-93.75	49.14 (1.76)
		(-28.29, 215.03)	(-233.63, 46.14)	53.03 (1.51)
	Normal BMI, females	0.19	14.23	46.49 (2.16)
		(-0.16, 0.55)	(-14.38, 42.83)	51.75 (2.78)
	Overweight/obese	128.38*	202.78	39.14 (6.74)
		(8.44, 248.33)	(-15.62, 421.19)	68.17 (7.93)
7. Additional control for Mediterranean diet score (Rhea only)	Normal BMI, males	-138.83	126.83	55.80 (2.01)
		(-584.74, 307.07)	(-378.27, 631.94)	51.63 (1.96)
	Normal BMI, females	-0.75	-11.55	50.43 (10.18)
		(-7.39, 5.90)	(-229.73, 206.64)	46.71 (12.32)
	Overweight/obese	51.05	136.07	47.80 (13.91)
		(-372.11, 474.21)	(-517.90, 790.04)	57.43 (15.52)

Note: a: values refer to predicted scores for the 5th and 95th percentiles of wGWG; all models were adjusted for maternal first trimester (MEFAB) or pre-pregnancy (Rhea) weight, maternal age at delivery, smoking and alcohol consumption during pregnancy, parent's level of education, parity, children's age at assessment and cohort. Children's sex was additionally controlled for in non-stratified models.

§: p<0.06; *: p<0.05; **: p<0.01

Table 11: Results of sensitivity analyses (outcome: internalizing behaviour)

Sensitivity analyses	Stratification group	wGWG estimates		Predicted scores (SE) range[a]
1. Gestational age >36 complete weeks	Normal BMI, males	-15.23<br>(-59.26, 28.79)	-10.67<br>(-34.95, 13.62)	63.94 (13.59)<br>41.44 (15.46)
	Normal BMI, females	26.84<br>(-42.97, 96.65)	95.81<br>(-67.81, 259.43)	44.73 (4.75)<br>55.07 (5.67)
	Overweight	117.27**<br>(32.82, 201.72)	219.76*<br>(51.27, 388.25)	37.75 (5.45)<br>69.41 (6.41)
2. Complete gestational weight data	Normal BMI, males	-8.54<br>(-36.84, 19.75)	-8.68<br>(-22.90, 5.55)	60.82 (8.72)<br>44.40 (9.74)
	Normal BMI, females	90.05*<br>(9.66, 170.55)	158.57§<br>(-2.97, 320.11)	40.85 (5.47)<br>65.54 (6.40)
	Overweight	90.05*<br>(9.66, 170.44)	158.57<br>(-2.97, 320.11)	40.85 (5.47)<br>65.54 (6.40)
3. Analyses by cohort	MEFAB, Normal BMI, males	-9.88<br>(-40.54, 20.77)	-9.84<br>(-25.03, 5.36)	62.71 (10.30)<br>42.03 (11.51)
	MEFAB, Normal BMI, females	25.44<br>(-55.11, 106.00)	87.16<br>(-108.14, 282.45)	44.62 (6.55)<br>55.63 (7.55)
	MEFAB, Overweight/Obese	89.84§<br>(-0.47, 180.15)	153.32<br>(-32.69, 339.33)	38.47 (7.85)<br>70.73 (9.86)
	Rhea, Normal BMI, males	-61.53<br>(-206.30, 83.24)	-28.33<br>(-112.14, 55.48)	71.12 (23.36)<br>34.62 (24.89)
	Rhea, Normal BMI, females	-243.00<br>(-837.75, 351.75)	-438.74<br>(-1616.58, 739.10)	63.44 (18.86)<br>32.85 (20.63)
	Rhea, Overweight/Obese	-126.88<br>(-565.07, 311.32)	-99.71<br>(-773.64, 574.22)	58.31 (14.37)<br>45.15 (16.03)
4. Additional control for breastfeeding and day-care attendance	Normal BMI, males	-14.97<br>(-42.35, 12.42)	-10.96<br>(-24.80, 2.88)	63.53 (8.04)<br>41.30 (8.86)
	Normal BMI, females	28.54<br>(-40.27, 97.35)	109.62<br>(-52.38, 271.62)	44.11 (4.66)<br>55.62 (5.59)
	Overweight/obese	95.77*<br>(16.43, 175.10)	182.70*<br>(24.90, 340.50)	40.27 (5.11)<br>66.31 (5.99)
5. Analyses excluding underweight and obese women	Normal BMI, males	-10.34<br>(-39.63, 18.95)	-9.32<br>(-23.96, 5.30)	60.99 (8.22)<br>44.35 (8.97)
	Normal BMI, females	22.04<br>(-47.72, 91.80)	84.73<br>(-79.49, 248.94)	45.24 (4.75)<br>54.19 (5.71)
	Overweight	115.79*<br>(14.56, 217.02)	267.80**<br>(68.66, 466.94)	35.89 (6.21)<br>69.73 (7.35)
6. Complete-case analyses	Normal BMI, males	-9.69<br>(-40.33, 20.95)	-9.34<br>(-24.45, 5.76)	61.19 (8.90)<br>44.21 (9.73)
	Normal BMI, females	42.53<br>(-30.90, 115.96)	108.12<br>(-67.49, 283.73)	42.81 (5.03)<br>56.10 (6.02)
	Overweight/obese	132.19*<br>(18.13, 246.24)	207.29*<br>(-0.39, 414.98)	38.13 (6.41)<br>67.92 (7.54)
7. Additional control for Mediterranean diet score (Rhea only)	Normal BMI, males	-44.29<br>(-190.98, 102.41)	-17.74<br>(-102.78, 67.30)	65.44 (23.68)<br>40.67 (25.24)
	Normal BMI, females	-231.86<br>(-828.11, 364.38)	-415.82<br>(-1596.56, 764.93)	62.72 (18.90)<br>33.63 (20.68)
	Overweight/obese	-111.37<br>(-559.15, 336.42)	-71.02<br>(-763.20, 621.15)	57.20 (14.72)<br>46.39 (16.42)

Note: a: values refer to predicted scores for the 5th and 95th percentiles of wGWG; all models were adjusted for maternal first trimester (MEFAB) or pre-pregnancy (Rhea) weight, maternal age at delivery, smoking and alcohol consumption during pregnancy, parent's level of education, parity, children's age at assessment and cohort. Children's sex was additionally controlled for in non-stratified models.

§: p<0.06; *: p<0.05; **: p<0.01

Table 12: Results of sensitivity analyses (outcome: externalizing behaviour)

**Sensitivity analyses**	**Stratification group**	**wGWG estimates**		**Predicted scores (SE) range[a]**
1. Gestational age >36 complete weeks	Normal BMI	-27.22*<br>(-54.44, -0.00)	-56.46<br>(-137.11, 24.19)	56.32 (2.53)<br>46.57 (3.24)
	Overweight/obese	71.43<br>(-16.02, 158.89)	150.46<br>(-23.87, 324.79)	44.74 (5.64)<br>65.28 (6.63)
2. Complete gestational weight data	Normal BMI	-25.74<br>(-53.07, 1.58)	-65.79<br>(-145.08, 13.50)	56.28 (2.63)<br>45.56 (3.41)
	Overweight/obese	69.78<br>(-17.71, 157.27)	161.36<br>(-14.47, 337.18)	43.78 (5.95)<br>65.94 (6.97)
3. Analyses by cohort	MEFAB, Normal BMI	-27.71§<br>(-56.36, 0.93)	-74.24<br>(-157.33, 8.85)	56.22 (3.18)<br>41.96 (4.41)
	MEFAB, Overweight/obese	70.42<br>(-36.12, 176.95)	168.46<br>(-51.10, 388.01)	39.87 (9.26)<br>69.78 (11.63)
	Rhea, Normal BMI	-58.76<br>(-191.73, 74.21)	-116.06<br>(-514.56, 282.43)	58.48 (6.97)<br>47.69 (7.97)
	Rhea, Overweight/obese	52.74<br>(-350.92, 456.41)	92.82<br>(-528.02, 713.66)	50.93 (13.24)<br>58.72 (14.15)
4. Additional control for breastfeeding and day-care attendance	Normal BMI	-28.37*<br>(-55.25, -1.50)	-63.07<br>(-141.36, 15.22)	56.47 (2.49)<br>45.87 (3.21)
	Overweight/obese	60.59<br>(-21.55, 142.73)	140.66<br>(-22.71, 304.03)	45.57 (5.29)<br>63.94 (6.21)
5. Analyses excluding underweight and obese women	Normal BMI	-23.96<br>(-52.08, 4.35)	-60.65<br>(-141.26, 19.97)	55.93 (2.56)<br>46.67 (3.24)
	Overweight	64.09<br>(-37.92, 166.11)	186.91<br>(-13.60, 387.41)	43.42 (6.26)<br>64.97 (7.41)
6. Complete-case analyses	Normal BMI	-12.58<br>(-43.94, 18.77)	-41.05<br>(-132.14, 50.03)	53.95 (2.92)<br>48.25 (3.73)
	Overweight/obese	97.21<br>(-19.05, 213.47)	159.89<br>(-51.81, 371.58)	44.05 (6.53)<br>66.48 (7.68)
7. Additional control for Mediterranean diet score (Rhea only)	Normal BMI	-51.58<br>(-185.43, 82.28)	-92.63<br>(-494.36, 309.10)	57.70 (7.02)<br>48.59 (8.03)
	Overweight/obese	87.77<br>(-322.20, 497.74)	154.79<br>(-479.06, 788.63)	48.48 (13.48)<br>61.46 (15.04)

Note: a: values refer to predicted scores for the 5th and 95th percentiles of wGWG; all models were adjusted for maternal first trimester (MEFAB) or pre-pregnancy (Rhea) weight, maternal age at delivery, smoking and alcohol consumption during pregnancy, parent's level of education, parity, children's sex, children's age at assessment and cohort.

§: $p<0.06$; *: $p<0.05$; **: $p<0.01$

Mediation analyses were performed using the PARAMED command in Stata 14 (1).

This method allows for the computation of the controlled direct effect (CDE), the natural direct effect (NDE), the natural indirect effect (NIE) and the marginal total effect (MTE).
CDE measures the expected increase in the outcome as the exposure changes and the mediator is fixed at a pre-specified level.
NDE measures the expected increase in the outcome when the changing the exposure (for example from X=0 to X=1) and keeping the mediator constant to the value it would have had if the exposure did not have changed.
NIE measures the expected increase in the outcome when the changing the mediator and keeping the exposure constant.
MTE is the sum of NIE and NDE. It measures the change in the outcome for a change in the exposure, considering also the effect of the mediator and controlling for the average level of the covariates (set at their average level).

Table 13: Results of mediation analyses (outcome: total problems)

Mediator	Stratification group	Controlled direct effect (SE)	Natural indirect effect (SE)	Marginal total effect (SE)
Gestational diabetes	Normal BMI	16.06 (19.54)	-1.05 (1.78)	-0.00 (398.34)
	Overweight	29.72 (17.17)	-4.22 (11.92)	18.90 (827.08)
Delivery mode	Normal BMI	-2.04 (1.84)	-0.13 (0.15)	0.37 (17.53)
	Overweight	22.47 (11.49)*	-2.74 (4.21)	18.93 (62.13)
Gestational age	Normal BMI	-0.12 (1.18)	-0.38 (0.26)	0.40 (1.24)
	Overweight	17.33 (8.33)*	0.00 (0.13)	17.62 (8.04)*
Birthweight	Normal BMI	-3.22 (3.37)	-0.02 (0.15)	-0.81 (1.36)
	Overweight	9.38 (9.84)	0.08 (1.05)	14.03 (8.27)
Child BMI	Normal BMI	0.03 (1.16)	-0.02 (0.09)	-0.56 (1.21)
	Overweight	15.54 (9.82)	0.43 (1.28)	16.85 (8.38)*

Note: all models were adjusted for maternal first trimester (MEFAB) or pre-pregnancy (Rhea) weight, maternal age at delivery, smoking and alcohol consumption during pregnancy, parent's level of education, parity, children's sex, children's age at assessment and cohort.

*: $p<0.05$

Table 14: Results of additional mediation analyses in the Rhea cohort (outcome: total problems)

Mediator	Stratification group	Controlled direct effect (SE)	Natural indirect effect (SE)	Marginal total effect (SE)
Cord-blood leptin	Normal BMI	-108.96 (65.73)	4.72 (13.58)	-105.01 (66.97)
	Overweight	16.01 (18.45)	-17.01 (16.70)	1.75 (22.02)
Leptin at 4 years	Normal BMI	-76.09 (55.40)	4.13 (13.51)	-76.26 (55.47)
	Overweight	16.28 (19.39)	0.99 (11.79)	19.24 (19.88)
Child TNFα	Normal BMI	-81.41 (53.10)	1.32 (17.58)	-83.50 (53.05)
	Overweight	20.10 (23.50)	0.50 (4.47)	16.74 (23.75)

Note: all models were adjusted for maternal pre-pregnancy weight, maternal age at delivery, smoking and alcohol consumption during pregnancy, parent's level of education, parity, children's sex and children's age at assessment.

Table 15: Results of mediation analyses (outcome: internalizing behaviour)

**Mediator**	**Stratification group**	**Controlled direct effect (SE)**	**Natural indirect effect (SE)**	**Marginal total effect (SE)**
Gestational diabetes	Normal BMI	26.48 (18.41)	-2.39 (4.98)	0.53 (293.89)
	Overweight	18.43 (16.32)	-1.72 (6.36)	16.05 (327.68)
Delivery mode	Normal BMI	-2.60 (1.81)	-0.22 (0.21)	0.35 (16.77)
	Overweight	14.16 (10.84)	0.43 (3.73)	15.34 (13.44)
Gestational age	Normal BMI	-0.30 (1.33)	-0.42 (0.29)	0.49 (1.42)
	Overweight	18.26 (7.83)*	-0.01 (0.69)	18.10 (7.57)*
Birthweight	Normal BMI	-4.63 (3.45)	0.16 (0.25)	-1.46 (1.55)
	Overweight	17.21 (9.38)	0.04 (0.46)	17.80 (7.89)*
Child BMI	Normal BMI	-0.39 (1.32)	-0.01 (0.03)	-1.14 (1.37)
	Overweight	16.12 (9.27)	0.09 (1.02)	16.72 (7.91)*

Note: all models were adjusted for maternal first trimester (MEFAB) or pre-pregnancy (Rhea) weight, maternal age at delivery, smoking and alcohol consumption during pregnancy, parent's level of education, parity, children's sex, children's age at assessment and cohort.
*: $p<0.05$; **: $p<0.01$

Table 16: Results of additional mediation analyses in the Rhea cohort (outcome: internalizing behaviour)

**Mediator**	**Stratification group**	**Controlled direct effect (SE)**	**Natural indirect effect (SE)**	**Marginal total effect (SE)**
Cord-blood leptin	Normal BMI	-69.27 (61.47)	1.21 (11.42)	-68.58 (62.67)
	Overweight	12.67 (24.99)	6.39 (15.73)	24.56 (26.36)
Leptin at 4 years	Normal BMI	-19.37 (49.16)	4.05 (13.15)	-18.98 (49.33)
	Overweight	18.62 (17.89)	15.02 (14.35)	20.55 (18.25)
Child TNFα	Normal BMI	-27.75 (47.59)	6.81 (16.56)	-29.96 (47.54)
	Overweight	-33.76 (57.81)	-2.37 (19.11)	-33.60 (59.14)

Note: all models were adjusted for maternal pre-pregnancy weight, maternal age at delivery, smoking and alcohol consumption during pregnancy, parent's level of education, parity, children's sex and children's age at assessment.

Table 17: Results of mediation analyses (outcome: externalizing behaviour)

Mediator	Stratification group	Controlled direct effect (SE)	Natural indirect effect (SE)	Marginal total effect (SE)
Gestational diabetes	Normal BMI	-0.83 (16.64)	-0.62 (2.77)	-1.95 (184.31)
	Overweight	22.21 (17.03)	-1.87 (6.78)	16.30 (371.51)
Delivery mode	Normal BMI	4.99 (6.62)	-0.09 (0.53)	-0.76 (59.56)
	Overweight	23.67 (11.22)*	-4.18 (4.47)	17.81 (95.17)
Gestational age	Normal BMI	1.91 (4.02)	0.01 (0.12)	-0.08 (3.48)
	Overweight	14.95 (8.16)	0.00 (0.44)	14.98 (7.87)
Birthweight	Normal BMI	-0.11 (4.13)	0.00 (0.17)	-1.89 (3.25)
	Overweight	5.13 (9.61)	0.10 (1.25)	10.05 (8.08)
Child BMI	Normal BMI	-2.47 (3.29)	0.10 (0.55)	-1.89 (3.26)
	Overweight	17.07 (9.58)	-0.57 (1.40)	15.97 (8.20)§

Note: all models were adjusted for maternal first trimester (MEFAB) or pre-pregnancy (Rhea) weight, maternal age at delivery, smoking and alcohol consumption during pregnancy, parent's level of education, parity, children's sex, children's age at assessment and cohort.

§: $p<0.06$; *: $p<0.05$

Table 18: Results of additional mediation analyses in the Rhea cohort (outcome: externalizing behaviour)

Mediator	Stratification group	Controlled direct effect (SE)	Natural indirect effect (SE)	Marginal total effect (SE)
Cord-blood leptin	Normal BMI	12.54 (12.96)	-0.51 (2.38)	12.42 (9.83)
	Overweight	15.98 (15.57)	-9.44 (12.21)	6.95 (19.11)
Leptin at 4 years	Normal BMI	10.80 (8.53)	-0.99 (1.85)	9.85 (8.53)
	Overweight	16.00 (18.23)	0.62 (7.38)	17.91 (18.48)
Child TNFα	Normal BMI	7.15 (8.01)	0.67 (1.94)	7.99 (7.69)
	Overweight	19.72 (22.78)	0.61 (5.37)	16.62 (22.95)

Note: all models were adjusted for maternal pre-pregnancy weight, maternal age at delivery, smoking and alcohol consumption during pregnancy, parent's level of education, parity, children's sex and children's age at assessment.

References:

1. Emsley R, Liu H. PARAMED: Stata module to perform causal mediation analysis using parametric regression models. Statistical Software Components. 2013.
